# Poly[di-μ-aqua-bis­(μ-2-amino-4-nitro­benzoato)dicaesium]

**DOI:** 10.1107/S1600536811026614

**Published:** 2011-07-09

**Authors:** Graham Smith, Urs D. Wermuth

**Affiliations:** a Faculty of Science and Technology, Queensland University of Technology, GPO Box 2434, Brisbane, Queensland 4001, Australia

## Abstract

In the structure of title compound, [Cs_2_(C_7_H_5_N_2_O_4_)_2_(H_2_O)_2_]_*n*_, the asymmetric unit contains two independent Cs atoms comprising different coordination polyhedra. One is nine-coordinate, the other seven-coordinate, both having irregular configurations. The CsO_9_ coordination polyhedron comprises O-atom donors from three bridging water mol­ecules, one of which is doubly bridging, three from carboxyl­ate groups, and three from nitro groups, of which two are bidentate chelate bridging. The CsO_6_N coordination polyhedron comprises the two bridging water mol­ecules, one amine N-atom donor, one carboxyl­ate O-atom donor and four O-atom donors from nitro groups (two from the chelate bridges). The extension of the dimeric unit gives a three-dimensional polymeric structure, which is stabilized by both intra- and inter­molecular amine N—H⋯O and water O—H⋯O hydrogen bonds to carboxyl­ate O-atom acceptors, as well as inter-ring π–π inter­actions [minimum ring centroid–centroid separation = 3.4172 (15) Å].

## Related literature

For the structures of some Cs complexes of aromatic carboxylic acids, see: Wiesbrock & Schmidbaur (2003 ▶); Hu *et al.* (2005 ▶); Smith & Wermuth (2010 ▶). For Lewis base salts of 4-nitro­anthranilic acid, see: Smith *et al.* (2002 ▶, 2004 ▶, 2007 ▶).
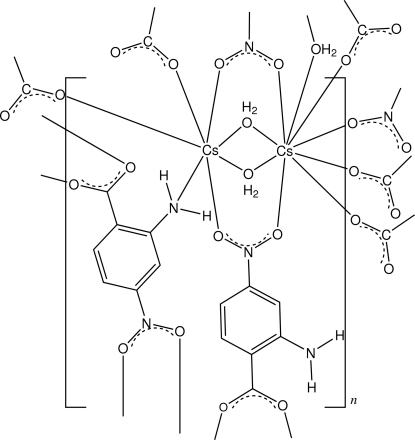


## Experimental

### 

#### Crystal data

[Cs_2_(C_7_H_5_N_2_O_4_)_2_(H_2_O)_2_]
*M* *_r_* = 664.12
Monoclinic, 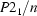

*a* = 15.3615 (3) Å
*b* = 6.9573 (2) Å
*c* = 18.3714 (4) Å
β = 97.903 (2)°
*V* = 1944.79 (8) Å^3^
*Z* = 4
Mo *K*α radiation
μ = 3.81 mm^−1^
*T* = 200 K
0.40 × 0.30 × 0.10 mm

#### Data collection

Oxford Diffraction Gemini-S CCD-detector diffractometer
Absorption correction: multi-scan (*CrysAlis PRO*; Oxford Diffraction, 2010 ▶) *T*_min_ = 0.411, *T*_max_ = 0.980
14204 measured reflections
4555 independent reflections
3818 reflections with *I* > 2σ(*I*)
*R*_int_ = 0.026

#### Refinement

*R*[*F* ^2^ > 2σ(*F* ^2^)] = 0.024
*wR*(*F* ^2^) = 0.049
*S* = 1.02
4555 reflections
303 parameters
H atoms treated by a mixture of independent and constrained refinement
Δρ_max_ = 0.47 e Å^−3^
Δρ_min_ = −0.90 e Å^−3^

### 

Data collection: *CrysAlis PRO* (Oxford Diffraction, 2010 ▶); cell refinement: *CrysAlis PRO*; data reduction: *CrysAlis PRO*; program(s) used to solve structure: *SIR92* (Altomare *et al.*, 1994 ▶); program(s) used to refine structure: *SHELXL97* (Sheldrick, 2008 ▶) within *WinGX* (Farrugia, 1999 ▶); molecular graphics: *PLATON* (Spek, 2009 ▶); software used to prepare material for publication: *PLATON*.

## Supplementary Material

Crystal structure: contains datablock(s) global, I. DOI: 10.1107/S1600536811026614/wm2509sup1.cif

Structure factors: contains datablock(s) I. DOI: 10.1107/S1600536811026614/wm2509Isup2.hkl

Additional supplementary materials:  crystallographic information; 3D view; checkCIF report

## Figures and Tables

**Table 1:** Selected bond lengths (Å)

Cs1—O1*W*	3.177 (2)
Cs1—O2*W*	3.311 (3)
Cs1—O42*A*	3.271 (2)
Cs1—O1*W*^i^	3.414 (3)
Cs1—O42*A*^i^	3.271 (2)
Cs1—O12*A*^ii^	3.165 (2)
Cs1—O11*B*^iii^	3.166 (2)
Cs1—O12*A*^iv^	3.202 (2)
Cs1—O41*B*^iv^	3.326 (2)
Cs2—O1*W*	3.248 (3)
Cs2—O2*W*	3.108 (3)
Cs2—O41*A*	3.136 (2)
Cs2—N2*B*	3.352 (3)
Cs2—O42*B*^v^	3.114 (2)
Cs2—O12*B*^vi^	3.090 (2)
Cs2—O42*B*^iv^	3.181 (2)

Symmetry codes: (i) 
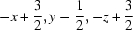
; (ii) 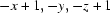; (iii) 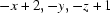; (iv) 
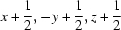
; (v) 
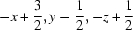
; (vi) 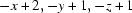.

**Table 2:** Hydrogen-bond geometry (Å, °)

*D*—H⋯*A*	*D*—H	H⋯*A*	*D*⋯*A*	*D*—H⋯*A*
N2*A*—H22*A*⋯O11*B*^v^	0.88 (3)	2.35 (3)	3.132 (3)	148 (3)
N2*A*—H21*A*⋯O12*A*	0.86 (4)	2.06 (4)	2.685 (4)	129 (3)
N2*B*—H21*B*⋯O11*A*^vii^	0.90 (3)	2.08 (3)	2.848 (3)	143 (3)
N2*B*—H22*B*⋯O12*B*	0.82 (3)	2.04 (3)	2.657 (4)	131 (3)
O1*W*—H11*W*⋯O11*B*^vi^	0.90 (5)	1.88 (4)	2.768 (3)	169 (3)
O1*W*—H12*W*⋯O12*A*^vii^	0.85 (4)	1.99 (4)	2.839 (3)	180 (5)
O2*W*—H21*W*⋯O11*A*^ii^	0.85 (4)	2.01 (4)	2.851 (4)	179 (6)
O2*W*—H22*W*⋯O12*B*^iii^	0.81 (4)	1.96 (4)	2.769 (4)	172 (4)

Symmetry codes: (ii) 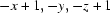; (iii) 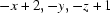; (v) 
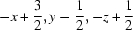
; (vi) 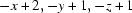; (vii) 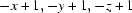.

**Fig. 1.**
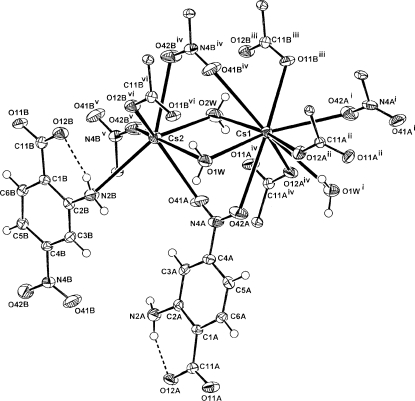
The molecular configuration and atom-numbering scheme for the dimeric repeat unit of (I), with non-H atoms drawn as 50% probability ellipsoids. For symmetry codes, see Table 1.

**Fig. 2.**
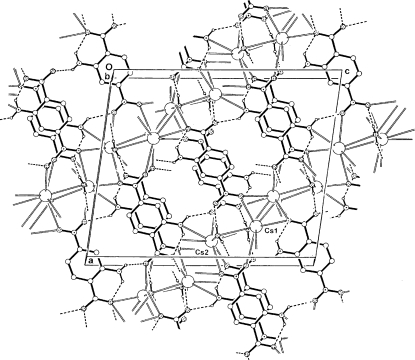
The polymeric structure of (I) in the unit cell viewed down b. Non-associative H atoms are omitted and hydrogen bonds are shown as dashed lines.

## supplementary crystallographic information

### Comment

The structures of the alkali metal complexes with aromatic carboxylic acids are of interest, particularly with the heavier metals Rb and Cs, because of their expanded coordination spheres and their ability to form polymeric systems. We obtained red crystals of the title compound [Cs_2_(C_7_H_5_N_2_O_4_)_2_(H_2_O)_2_]_n_ (I) from the reaction of caesium carbonate with 4-nitroanthranilic acid (4-NAA), and the structure is reported here. Although the structure of the Cs complex with anthranilic acid has been reported (a 1:1 metal complex–acid adduct polymer) (Wiesbrock & Schmidbaur, 2003), no metal complexes of 4-nitroanthranilic are known. We have reported the structures of the 4-NAA salts of the Lewis bases ethylenediamine (a dihydrate) (Smith *et al.*, 2002), dicyclohexamine (anhydrous) (Smith *et al.*, 2004) and guanidine (a monohydrate) (Smith *et al.*, 2007).

In the structure of (I), the asymmetric unit contains two independent Cs atoms, one nine-coordinate, the other seven-coordinate, with both having irregular configurations (Fig. 1). The CsO_9_ coordination polyhedron about Cs1 comprises oxygen donors from three bridging water molecules, two of which bridge Cs1 and Cs2, three from carboxylate groups, and two from bidentate bridging nitro groups [Cs—O range, 3.165 (2)–3.414 (3) Å]. The CsO_6_N coordination polyhedron about Cs2 comprises the two bridging water atoms, one amine N donor, one carboxyl O donor and four O donors from nitro groups (two bidentate bridging) [Cs—O/(N) range, 3.090 (2)–3.352 (3) Å] (Table 1). The extension of the dimeric unit gives a three-dimensional polymeric structure (Fig. 2) which is stabilized by both intra- and intermolecular amine N—H···O and water O—H···O hydrogen bonds to only carboxyl O acceptors (Table 2). Also, there are inter-ring π–π interactions involving both ring 1 (C1A–C6A) and ring 2 (ring 1B–C6B): minimum centroid separation: rings 1–1^vii^, 3.4172 (15) Å; rings 2–2^v^, 3.6081 (16) Å (for symmetry codes, see Tables 1, 2).

These structural features, including expanded coordination spheres, multiple bridging and polymeric extensions are similar to those found in other Cs complexes with nitro-substituted aromatic carboxylates, *e.g.* cesium 3,5-dinitrosalicylate (Hu *et al.*, 2005) and cesium 5-nitroisophthalate (Smith & Wermuth, 2010).

### Experimental

The title compound was synthesized by heating together under reflux for 15 minutes, 1 mmol of caesium carbonate and 2 mmol of 4-nitroanthranilic acid in 50 ml of 1:4 ethanol–water. After concentration to *ca* 30 ml, partial room temperature evaporation of the hot-filtered solution gave flat red prisms of (I) from which a suitable specimen was cleaved for the X-ray analysis.

### Refinement

The amine and water H atoms were located in a difference-Fourier analysis and their positional and isotropic displacenemt parameters were refined. Other hydrogen atoms were included in the refinement in calculated positions with C—H = 0.93 Å and allowed to ride, with *U*_iso_(H) = 1.2*U*_eq_(C).

### Crystal data

**Table:**

[Cs_2_(C_7_H_5_N_2_O_4_)_2_(H_2_O)_2_]	*F*(000) = 1264
*M**_r_* = 664.12	*D*_x_ = 2.268 Mg m^−^^3^
Monoclinic, *P*2_1_/*n*	Mo *K*α radiation, λ = 0.71073 Å
Hall symbol: -P 2yn	Cell parameters from 8867 reflections
*a* = 15.3615 (3) Å	θ = 3.2–28.7°
*b* = 6.9573 (2) Å	µ = 3.81 mm^−^^1^
*c* = 18.3714 (4) Å	*T* = 200 K
β = 97.903 (2)°	Plate, red
*V* = 1944.79 (8) Å^3^	0.40 × 0.30 × 0.10 mm
*Z* = 4	

### Data collection

**Table:**

Oxford Diffraction Gemini-S CCD-detector diffractometer	4555 independent reflections
Radiation source: Enhance (Mo) X-ray source	3818 reflections with *I* > 2σ(*I*)
graphite	*R*_int_ = 0.026
Detector resolution: 16.077 pixels mm^-1^	θ_max_ = 28.8°, θ_min_ = 3.3°
ω scans	*h* = −20→20
Absorption correction: multi-scan (*CrysAlis PRO*; Oxford Diffraction, 2010)	*k* = −8→9
*T*_min_ = 0.411, *T*_max_ = 0.980	*l* = −23→23
14204 measured reflections	

### Refinement

**Table:**

Refinement on *F*^2^	Primary atom site location: structure-invariant direct methods
Least-squares matrix: full	Secondary atom site location: difference Fourier map
*R*[*F*^2^ > 2σ(*F*^2^)] = 0.024	Hydrogen site location: inferred from neighbouring sites
*wR*(*F*^2^) = 0.049	H atoms treated by a mixture of independent and constrained refinement
*S* = 1.02	*w* = 1/[σ^2^(*F*_o_^2^) + (0.0232*P*)^2^] where *P* = (*F*_o_^2^ + 2*F*_c_^2^)/3
4555 reflections	(Δ/σ)_max_ = 0.002
303 parameters	Δρ_max_ = 0.47 e Å^−^^3^
0 restraints	Δρ_min_ = −0.90 e Å^−^^3^

### Special details

**Table:**

Geometry. Bond distances, angles *etc*. have been calculated using the rounded fractional coordinates. All su's are estimated from the variances of the (full) variance-covariance matrix. The cell e.s.d.'s are taken into account in the estimation of distances, angles and torsion angles
Refinement. Refinement of *F*^2^ against ALL reflections. The weighted *R*-factor *wR* and goodness of fit *S* are based on *F*^2^, conventional *R*-factors *R* are based on *F*, with *F* set to zero for negative *F*^2^. The threshold expression of *F*^2^ > σ(*F*^2^) is used only for calculating *R*-factors(gt) *etc*. and is not relevant to the choice of reflections for refinement. *R*-factors based on *F*^2^ are statistically about twice as large as those based on *F*, and *R*- factors based on ALL data will be even larger.

### Fractional atomic coordinates and isotropic or equivalent isotropic displacement parameters (Å^2^)

**Table:**

	*x*	*y*	*z*	*U*_iso_*/*U*_eq_	
Cs1	0.84171 (1)	−0.10346 (2)	0.72068 (1)	0.0226 (1)	
Cs2	0.90529 (1)	0.22312 (3)	0.53616 (1)	0.0262 (1)	
O1W	0.83714 (17)	0.3457 (3)	0.68930 (14)	0.0301 (8)	
O2W	0.8960 (2)	−0.2190 (4)	0.55795 (16)	0.0418 (9)	
O11A	0.26335 (13)	0.4294 (3)	0.47800 (11)	0.0320 (7)	
O11B	1.01545 (12)	0.4344 (3)	0.26444 (11)	0.0278 (7)	
O12A	0.29653 (13)	0.3889 (3)	0.36507 (11)	0.0274 (6)	
O12B	0.97730 (13)	0.4711 (3)	0.37664 (11)	0.0308 (7)	
O41A	0.71088 (13)	0.0800 (3)	0.53048 (14)	0.0398 (8)	
O41B	0.54158 (14)	0.4375 (4)	0.20587 (13)	0.0476 (9)	
O42A	0.66924 (15)	0.1204 (3)	0.63677 (13)	0.0404 (8)	
O42B	0.58798 (14)	0.4656 (4)	0.10161 (12)	0.0451 (8)	
N2A	0.45730 (19)	0.2618 (4)	0.34723 (15)	0.0282 (8)	
N2B	0.8077 (2)	0.4733 (4)	0.39156 (14)	0.0308 (9)	
N4A	0.65627 (16)	0.1286 (3)	0.56911 (16)	0.0282 (8)	
N4B	0.60057 (15)	0.4527 (3)	0.16866 (14)	0.0254 (7)	
C1A	0.40690 (16)	0.3164 (4)	0.46641 (14)	0.0161 (8)	
C1B	0.86323 (17)	0.4523 (4)	0.27425 (15)	0.0177 (8)	
C2A	0.47163 (18)	0.2593 (3)	0.42261 (15)	0.0183 (8)	
C2B	0.79362 (17)	0.4629 (4)	0.31685 (15)	0.0195 (8)	
C3A	0.55438 (17)	0.2026 (4)	0.45856 (16)	0.0206 (8)	
C3B	0.70712 (18)	0.4587 (4)	0.28022 (15)	0.0208 (8)	
C4A	0.56969 (17)	0.1972 (4)	0.53363 (16)	0.0208 (8)	
C4B	0.69270 (17)	0.4511 (4)	0.20513 (15)	0.0190 (8)	
C5A	0.50738 (18)	0.2486 (3)	0.57821 (16)	0.0210 (8)	
C5B	0.75952 (18)	0.4438 (4)	0.16125 (15)	0.0216 (8)	
C6A	0.42732 (17)	0.3090 (4)	0.54248 (15)	0.0192 (8)	
C6B	0.84406 (17)	0.4416 (4)	0.19793 (15)	0.0200 (8)	
C11A	0.31553 (17)	0.3831 (4)	0.43413 (15)	0.0190 (8)	
C11B	0.95942 (18)	0.4511 (4)	0.30752 (16)	0.0219 (8)	
H3B	0.65990	0.46120	0.30690	0.0250*	
H5B	0.74790	0.44050	0.11020	0.0260*	
H21B	0.764 (2)	0.501 (4)	0.4175 (17)	0.030 (9)*	
H3A	0.59870	0.16870	0.43130	0.0250*	
H22B	0.857 (2)	0.507 (4)	0.4101 (17)	0.029 (9)*	
H5A	0.51900	0.24270	0.62920	0.0250*	
H6A	0.38460	0.34700	0.57080	0.0230*	
H6B	0.89050	0.43270	0.17050	0.0240*	
H11W	0.888 (3)	0.411 (6)	0.699 (2)	0.084 (16)*	
H12W	0.797 (3)	0.425 (5)	0.673 (2)	0.048 (14)*	
H21A	0.404 (3)	0.271 (5)	0.326 (2)	0.059 (13)*	
H21W	0.849 (3)	−0.282 (5)	0.547 (2)	0.037 (13)*	
H22A	0.487 (2)	0.182 (5)	0.3227 (18)	0.039 (10)*	
H22W	0.932 (3)	−0.289 (6)	0.581 (2)	0.056 (13)*	

### Atomic displacement parameters (Å^2^)

**Table:**

	*U*^11^	*U*^22^	*U*^33^	*U*^12^	*U*^13^	*U*^23^
Cs1	0.0213 (1)	0.0241 (1)	0.0224 (1)	0.0021 (1)	0.0035 (1)	−0.0006 (1)
Cs2	0.0251 (1)	0.0282 (1)	0.0242 (1)	−0.0013 (1)	−0.0008 (1)	−0.0002 (1)
O1W	0.0246 (13)	0.0298 (12)	0.0338 (14)	−0.0002 (11)	−0.0029 (10)	0.0040 (11)
O2W	0.0317 (15)	0.0376 (14)	0.0520 (18)	−0.0006 (13)	−0.0084 (13)	0.0074 (12)
O11A	0.0207 (10)	0.0497 (13)	0.0255 (12)	0.0087 (10)	0.0024 (9)	−0.0078 (10)
O11B	0.0145 (10)	0.0370 (12)	0.0316 (12)	0.0014 (9)	0.0022 (9)	−0.0027 (9)
O12A	0.0236 (10)	0.0377 (12)	0.0198 (11)	0.0084 (9)	−0.0007 (8)	0.0013 (9)
O12B	0.0235 (11)	0.0430 (12)	0.0241 (11)	0.0009 (10)	−0.0035 (9)	−0.0032 (10)
O41A	0.0172 (11)	0.0420 (14)	0.0602 (17)	0.0041 (10)	0.0050 (11)	−0.0026 (12)
O41B	0.0195 (11)	0.0811 (19)	0.0434 (15)	0.0017 (12)	0.0087 (11)	0.0026 (13)
O42A	0.0311 (12)	0.0438 (13)	0.0408 (15)	0.0044 (10)	−0.0141 (11)	−0.0011 (11)
O42B	0.0272 (12)	0.0750 (17)	0.0295 (13)	−0.0086 (12)	−0.0086 (10)	0.0112 (12)
N2A	0.0280 (15)	0.0360 (15)	0.0216 (14)	0.0063 (12)	0.0074 (12)	−0.0002 (12)
N2B	0.0230 (14)	0.0484 (17)	0.0210 (14)	0.0039 (13)	0.0027 (12)	−0.0008 (12)
N4A	0.0173 (12)	0.0200 (12)	0.0448 (17)	−0.0038 (10)	−0.0050 (12)	−0.0019 (12)
N4B	0.0194 (12)	0.0280 (12)	0.0280 (14)	0.0009 (10)	0.0006 (11)	0.0016 (11)
C1A	0.0161 (13)	0.0131 (12)	0.0187 (14)	0.0014 (10)	0.0009 (10)	−0.0018 (10)
C1B	0.0181 (13)	0.0151 (12)	0.0193 (14)	0.0014 (11)	0.0005 (11)	−0.0011 (11)
C2A	0.0211 (14)	0.0120 (13)	0.0223 (15)	−0.0008 (10)	0.0049 (11)	0.0004 (10)
C2B	0.0216 (14)	0.0184 (12)	0.0184 (14)	0.0012 (11)	0.0024 (11)	0.0004 (11)
C3A	0.0170 (14)	0.0163 (13)	0.0300 (16)	−0.0024 (11)	0.0083 (11)	−0.0021 (12)
C3B	0.0181 (14)	0.0212 (13)	0.0241 (15)	−0.0004 (11)	0.0062 (11)	0.0004 (12)
C4A	0.0151 (13)	0.0136 (12)	0.0320 (17)	−0.0027 (11)	−0.0031 (12)	−0.0004 (12)
C4B	0.0140 (13)	0.0173 (12)	0.0247 (15)	−0.0007 (11)	−0.0011 (11)	0.0009 (11)
C5A	0.0238 (15)	0.0182 (14)	0.0197 (15)	−0.0023 (11)	−0.0017 (12)	0.0002 (11)
C5B	0.0222 (14)	0.0246 (14)	0.0176 (14)	−0.0023 (12)	0.0009 (11)	0.0003 (12)
C6A	0.0181 (13)	0.0193 (13)	0.0211 (15)	0.0008 (11)	0.0056 (11)	−0.0017 (11)
C6B	0.0209 (14)	0.0193 (13)	0.0208 (14)	−0.0004 (11)	0.0061 (11)	−0.0025 (11)
C11A	0.0190 (14)	0.0162 (13)	0.0217 (15)	−0.0002 (11)	0.0025 (11)	−0.0016 (11)
C11B	0.0216 (14)	0.0189 (13)	0.0248 (16)	−0.0016 (11)	0.0021 (12)	−0.0001 (12)

### Geometric parameters (Å, °)

**Table:**

Cs1—O1W	3.177 (2)	N2A—C2A	1.372 (4)
Cs1—O2W	3.311 (3)	N2B—C2B	1.362 (4)
Cs1—O42A	3.271 (2)	N4A—C4A	1.478 (4)
Cs1—O1W^i^	3.414 (3)	N4B—C4B	1.480 (4)
Cs1—O42A^i^	3.271 (2)	N2A—H21A	0.86 (4)
Cs1—O12A^ii^	3.165 (2)	N2A—H22A	0.88 (3)
Cs1—O11B^iii^	3.166 (2)	N2B—H21B	0.90 (3)
Cs1—O12A^iv^	3.202 (2)	N2B—H22B	0.82 (3)
Cs1—O41B^iv^	3.326 (2)	C1A—C2A	1.419 (4)
Cs2—O1W	3.248 (3)	C1A—C6A	1.391 (4)
Cs2—O2W	3.108 (3)	C1A—C11A	1.519 (4)
Cs2—O41A	3.136 (2)	C1B—C2B	1.411 (4)
Cs2—N2B	3.352 (3)	C1B—C6B	1.395 (4)
Cs2—O42B^v^	3.114 (2)	C1B—C11B	1.519 (4)
Cs2—O12B^vi^	3.090 (2)	C2A—C3A	1.406 (4)
Cs2—O42B^iv^	3.181 (2)	C2B—C3B	1.405 (4)
O11A—C11A	1.254 (3)	C3A—C4A	1.367 (4)
O11B—C11B	1.252 (3)	C3B—C4B	1.368 (4)
O12A—C11A	1.263 (3)	C4A—C5A	1.389 (4)
O12B—C11B	1.269 (4)	C4B—C5B	1.391 (4)
O41A—N4A	1.219 (3)	C5A—C6A	1.378 (4)
O41B—N4B	1.212 (3)	C5B—C6B	1.379 (4)
O42A—N4A	1.233 (4)	C3A—H3A	0.9300
O42B—N4B	1.224 (3)	C3B—H3B	0.9300
O1W—H11W	0.90 (5)	C5A—H5A	0.9300
O1W—H12W	0.85 (4)	C5B—H5B	0.9300
O2W—H21W	0.85 (4)	C6A—H6A	0.9300
O2W—H22W	0.81 (4)	C6B—H6B	0.9300
			
O1W—Cs1—O2W	94.42 (7)	Cs2^viii^—O42B—N4B	111.06 (17)
O1W—Cs1—O42A	56.62 (6)	Cs2^ix^—O42B—Cs2^viii^	93.23 (6)
O1W—Cs1—O1W^i^	100.99 (6)	Cs1—O1W—H12W	134 (3)
O1W—Cs1—O42A^i^	136.12 (6)	Cs2—O1W—H11W	86 (2)
O1W—Cs1—O12A^ii^	122.03 (6)	Cs2—O1W—H12W	100 (3)
O1W—Cs1—O11B^iii^	136.78 (6)	H11W—O1W—H12W	108 (4)
O1W—Cs1—O12A^iv^	71.93 (6)	Cs1^vii^—O1W—H11W	126 (3)
O1W—Cs1—O41B^iv^	68.98 (7)	Cs1^vii^—O1W—H12W	61 (3)
O2W—Cs1—O42A	88.76 (7)	Cs1—O1W—H11W	118 (3)
O1W^i^—Cs1—O2W	137.01 (7)	Cs2—O2W—H21W	122 (3)
O2W—Cs1—O42A^i^	128.85 (6)	Cs2—O2W—H22W	128 (3)
O2W—Cs1—O12A^ii^	68.49 (6)	H21W—O2W—H22W	108 (4)
O2W—Cs1—O11B^iii^	69.14 (6)	Cs1—O2W—H21W	92 (3)
O2W—Cs1—O12A^iv^	166.24 (6)	Cs1—O2W—H22W	85 (3)
O2W—Cs1—O41B^iv^	70.16 (7)	Cs2—N2B—C2B	139.7 (2)
O1W^i^—Cs1—O42A	67.92 (6)	O42A—N4A—C4A	118.1 (2)
O42A—Cs1—O42A^i^	121.97 (6)	O41A—N4A—C4A	118.9 (3)
O12A^ii^—Cs1—O42A	67.64 (5)	O41A—N4A—O42A	123.1 (3)
O11B^iii^—Cs1—O42A	153.35 (5)	O41B—N4B—C4B	119.1 (2)
O12A^iv^—Cs1—O42A	85.11 (5)	O41B—N4B—O42B	123.2 (2)
O41B^iv^—Cs1—O42A	119.55 (6)	O42B—N4B—C4B	117.7 (2)
O1W^i^—Cs1—O42A^i^	54.41 (6)	C2A—N2A—H21A	118 (3)
O1W^i^—Cs1—O12A^ii^	69.30 (5)	H21A—N2A—H22A	110 (3)
O1W^i^—Cs1—O11B^iii^	118.49 (5)	C2A—N2A—H22A	119 (2)
O1W^i^—Cs1—O12A^iv^	50.69 (5)	Cs2—N2B—H21B	88.6 (19)
O1W^i^—Cs1—O41B^iv^	152.73 (6)	C2B—N2B—H21B	121 (2)
O12A^ii^—Cs1—O42A^i^	85.71 (5)	C2B—N2B—H22B	116 (2)
O11B^iii^—Cs1—O42A^i^	67.36 (5)	H21B—N2B—H22B	116 (3)
O12A^iv^—Cs1—O42A^i^	64.51 (5)	Cs2—N2B—H22B	62 (2)
O41B^iv^—Cs1—O42A^i^	114.78 (6)	C6A—C1A—C11A	118.2 (2)
O11B^iii^—Cs1—O12A^ii^	89.79 (5)	C2A—C1A—C6A	118.7 (2)
O12A^ii^—Cs1—O12A^iv^	119.86 (5)	C2A—C1A—C11A	123.1 (2)
O12A^ii^—Cs1—O41B^iv^	137.81 (6)	C2B—C1B—C11B	123.1 (2)
O11B^iii^—Cs1—O12A^iv^	119.61 (5)	C6B—C1B—C11B	117.6 (2)
O11B^iii^—Cs1—O41B^iv^	67.85 (6)	C2B—C1B—C6B	119.3 (2)
O12A^iv^—Cs1—O41B^iv^	102.33 (6)	N2A—C2A—C1A	122.8 (3)
O1W—Cs2—O2W	97.03 (7)	N2A—C2A—C3A	119.1 (3)
O1W—Cs2—O41A	72.14 (6)	C1A—C2A—C3A	118.1 (2)
O1W—Cs2—N2B	112.82 (6)	N2B—C2B—C1B	122.3 (3)
O1W—Cs2—O42B^v^	154.92 (6)	C1B—C2B—C3B	118.2 (2)
O1W—Cs2—O12B^vi^	66.73 (6)	N2B—C2B—C3B	119.5 (3)
O1W—Cs2—O42B^iv^	98.84 (6)	C2A—C3A—C4A	120.0 (3)
O2W—Cs2—O41A	68.26 (7)	C2B—C3B—C4B	119.7 (3)
O2W—Cs2—N2B	126.34 (7)	C3A—C4A—C5A	123.4 (3)
O2W—Cs2—O42B^v^	62.78 (7)	N4A—C4A—C3A	118.3 (2)
O2W—Cs2—O12B^vi^	130.43 (7)	N4A—C4A—C5A	118.3 (3)
O2W—Cs2—O42B^iv^	66.20 (7)	C3B—C4B—C5B	123.8 (3)
O41A—Cs2—N2B	79.54 (7)	N4B—C4B—C5B	118.3 (2)
O41A—Cs2—O42B^v^	85.88 (6)	N4B—C4B—C3B	117.9 (2)
O12B^vi^—Cs2—O41A	136.14 (6)	C4A—C5A—C6A	116.1 (3)
O41A—Cs2—O42B^iv^	131.93 (6)	C4B—C5B—C6B	116.0 (2)
O42B^v^—Cs2—N2B	73.49 (7)	C1A—C6A—C5A	123.6 (3)
O12B^vi^—Cs2—N2B	102.64 (6)	C1B—C6B—C5B	123.1 (2)
O42B^iv^—Cs2—N2B	142.10 (7)	O11A—C11A—O12A	123.9 (3)
O12B^vi^—Cs2—O42B^v^	137.32 (6)	O11A—C11A—C1A	117.7 (2)
O42B^v^—Cs2—O42B^iv^	86.77 (6)	O12A—C11A—C1A	118.4 (2)
O12B^vi^—Cs2—O42B^iv^	70.52 (6)	O11B—C11B—C1B	117.5 (2)
Cs1—O1W—Cs2	84.10 (5)	O12B—C11B—C1B	117.8 (2)
Cs1—O1W—Cs1^vii^	90.76 (6)	O11B—C11B—O12B	124.7 (3)
Cs1^vii^—O1W—Cs2	145.43 (8)	C2A—C3A—H3A	120.00
Cs1—O2W—Cs2	84.13 (7)	C4A—C3A—H3A	120.00
Cs1^iii^—O11B—C11B	122.77 (17)	C2B—C3B—H3B	120.00
Cs1^ii^—O12A—C11A	121.78 (17)	C4B—C3B—H3B	120.00
Cs1^viii^—O12A—C11A	143.20 (18)	C4A—C5A—H5A	122.00
Cs1^ii^—O12A—Cs1^viii^	95.00 (5)	C6A—C5A—H5A	122.00
Cs2^vi^—O12B—C11B	128.65 (17)	C4B—C5B—H5B	122.00
Cs2—O41A—N4A	128.40 (18)	C6B—C5B—H5B	122.00
Cs1^viii^—O41B—N4B	141.79 (19)	C1A—C6A—H6A	118.00
Cs1—O42A—N4A	120.36 (17)	C5A—C6A—H6A	118.00
Cs1—O42A—Cs1^vii^	91.71 (6)	C1B—C6B—H6B	118.00
Cs1^vii^—O42A—N4A	139.56 (16)	C5B—C6B—H6B	118.00
Cs2^ix^—O42B—N4B	147.4 (2)		
			
O2W—Cs1—O1W—Cs2	−4.20 (7)	O1W—Cs2—N2B—C2B	−161.7 (3)
O2W—Cs1—O1W—Cs1^vii^	141.55 (7)	O2W—Cs2—N2B—C2B	−43.4 (3)
O42A—Cs1—O1W—Cs2	−89.79 (7)	O41A—Cs2—N2B—C2B	−96.3 (3)
O42A—Cs1—O1W—Cs1^vii^	55.96 (6)	O42B^v^—Cs2—N2B—C2B	−7.5 (3)
O1W^i^—Cs1—O1W—Cs2	−143.87 (6)	O12B^vi^—Cs2—N2B—C2B	128.5 (3)
O1W^i^—Cs1—O1W—Cs1^vii^	1.88 (7)	O42B^iv^—Cs2—N2B—C2B	54.0 (4)
O42A^i^—Cs1—O1W—Cs2	166.94 (6)	O1W—Cs2—O42B^v^—N4B^v^	116.9 (3)
O42A^i^—Cs1—O1W—Cs1^vii^	−47.32 (10)	O1W—Cs2—O42B^v^—Cs2^iii^	−104.03 (13)
O12A^ii^—Cs1—O1W—Cs2	−71.52 (7)	O2W—Cs2—O42B^v^—N4B^v^	156.1 (3)
O12A^ii^—Cs1—O1W—Cs1^vii^	74.23 (7)	O2W—Cs2—O42B^v^—Cs2^iii^	−64.82 (8)
O11B^iii^—Cs1—O1W—Cs2	59.73 (9)	O41A—Cs2—O42B^v^—N4B^v^	88.5 (3)
O11B^iii^—Cs1—O1W—Cs1^vii^	−154.52 (6)	O41A—Cs2—O42B^v^—Cs2^iii^	−132.46 (7)
O12A^iv^—Cs1—O1W—Cs2	174.05 (7)	N2B—Cs2—O42B^v^—N4B^v^	8.2 (3)
O12A^iv^—Cs1—O1W—Cs1^vii^	−40.21 (5)	N2B—Cs2—O42B^v^—Cs2^iii^	147.25 (8)
O41B^iv^—Cs1—O1W—Cs2	62.57 (6)	O1W—Cs2—O12B^vi^—C11B^vi^	33.6 (2)
O41B^iv^—Cs1—O1W—Cs1^vii^	−151.68 (7)	O2W—Cs2—O12B^vi^—C11B^vi^	−45.3 (2)
O1W—Cs1—O2W—Cs2	4.39 (7)	O41A—Cs2—O12B^vi^—C11B^vi^	55.3 (2)
O42A—Cs1—O2W—Cs2	60.77 (6)	N2B—Cs2—O12B^vi^—C11B^vi^	143.3 (2)
O1W^i^—Cs1—O2W—Cs2	115.69 (8)	O1W—Cs2—O42B^iv^—Cs2^iii^	155.41 (6)
O42A^i^—Cs1—O2W—Cs2	−167.73 (5)	O1W—Cs2—O42B^iv^—N4B^iv^	−2.3 (2)
O12A^ii^—Cs1—O2W—Cs2	127.16 (8)	O2W—Cs2—O42B^iv^—Cs2^iii^	61.58 (7)
O11B^iii^—Cs1—O2W—Cs2	−134.45 (8)	O2W—Cs2—O42B^iv^—N4B^iv^	−96.2 (2)
O41B^iv^—Cs1—O2W—Cs2	−61.39 (7)	O41A—Cs2—O42B^iv^—Cs2^iii^	81.52 (9)
O1W—Cs1—O42A—N4A	94.23 (19)	O41A—Cs2—O42B^iv^—N4B^iv^	−76.2 (2)
O1W—Cs1—O42A—Cs1^vii^	−59.92 (6)	N2B—Cs2—O42B^iv^—Cs2^iii^	−57.61 (13)
O2W—Cs1—O42A—N4A	−1.89 (19)	N2B—Cs2—O42B^iv^—N4B^iv^	144.65 (17)
O2W—Cs1—O42A—Cs1^vii^	−156.03 (6)	Cs1^iii^—O11B—C11B—O12B	57.9 (3)
O1W^i^—Cs1—O42A—N4A	−144.9 (2)	Cs1^iii^—O11B—C11B—C1B	−123.6 (2)
O1W^i^—Cs1—O42A—Cs1^vii^	60.99 (5)	Cs1^ii^—O12A—C11A—O11A	−68.7 (3)
O42A^i^—Cs1—O42A—N4A	−138.46 (18)	Cs1^ii^—O12A—C11A—C1A	111.4 (2)
O42A^i^—Cs1—O42A—Cs1^vii^	67.40 (7)	Cs1^viii^—O12A—C11A—O11A	113.3 (3)
O12A^ii^—Cs1—O42A—N4A	−69.08 (18)	Cs1^viii^—O12A—C11A—C1A	−66.7 (4)
O12A^ii^—Cs1—O42A—Cs1^vii^	136.78 (7)	Cs2^vi^—O12B—C11B—O11B	55.1 (4)
O11B^iii^—Cs1—O42A—N4A	−35.0 (3)	Cs2^vi^—O12B—C11B—C1B	−123.3 (2)
O11B^iii^—Cs1—O42A—Cs1^vii^	170.83 (9)	Cs2—O41A—N4A—O42A	−54.8 (3)
O12A^iv^—Cs1—O42A—N4A	165.78 (19)	Cs2—O41A—N4A—C4A	126.4 (2)
O12A^iv^—Cs1—O42A—Cs1^vii^	11.64 (5)	Cs1^viii^—O41B—N4B—O42B	−40.7 (4)
O41B^iv^—Cs1—O42A—N4A	64.4 (2)	Cs1^viii^—O41B—N4B—C4B	140.9 (2)
O41B^iv^—Cs1—O42A—Cs1^vii^	−89.77 (7)	Cs1—O42A—N4A—O41A	−10.5 (3)
O1W—Cs1—O1W^i^—Cs1^i^	161.87 (6)	Cs1—O42A—N4A—C4A	168.30 (16)
O1W—Cs1—O1W^i^—Cs2^i^	−117.46 (12)	Cs1^vii^—O42A—N4A—O41A	127.3 (2)
O2W—Cs1—O1W^i^—Cs1^i^	53.01 (11)	Cs1^vii^—O42A—N4A—C4A	−53.9 (4)
O2W—Cs1—O1W^i^—Cs2^i^	133.68 (12)	Cs2^ix^—O42B—N4B—O41B	110.6 (3)
O42A—Cs1—O1W^i^—Cs1^i^	115.00 (7)	Cs2^ix^—O42B—N4B—C4B	−70.9 (4)
O42A—Cs1—O1W^i^—Cs2^i^	−164.33 (14)	Cs2^viii^—O42B—N4B—O41B	−24.9 (3)
O1W—Cs1—O42A^i^—Cs1^i^	121.80 (8)	Cs2^viii^—O42B—N4B—C4B	153.57 (17)
O1W—Cs1—O42A^i^—N4A^i^	−22.7 (3)	Cs2—N2B—C2B—C1B	−58.5 (4)
O2W—Cs1—O42A^i^—Cs1^i^	−69.58 (9)	Cs2—N2B—C2B—C3B	120.3 (3)
O2W—Cs1—O42A^i^—N4A^i^	145.9 (3)	O41A—N4A—C4A—C3A	0.2 (4)
O42A—Cs1—O42A^i^—Cs1^i^	48.46 (7)	O41A—N4A—C4A—C5A	178.8 (2)
O42A—Cs1—O42A^i^—N4A^i^	−96.1 (3)	O42A—N4A—C4A—C3A	−178.6 (2)
O1W—Cs1—O12A^ii^—Cs1^i^	−131.61 (6)	O42A—N4A—C4A—C5A	−0.1 (4)
O1W—Cs1—O12A^ii^—C11A^ii^	47.2 (2)	O41B—N4B—C4B—C3B	−8.7 (4)
O2W—Cs1—O12A^ii^—Cs1^i^	146.96 (8)	O41B—N4B—C4B—C5B	171.9 (3)
O2W—Cs1—O12A^ii^—C11A^ii^	−34.2 (2)	O42B—N4B—C4B—C3B	172.7 (3)
O42A—Cs1—O12A^ii^—Cs1^i^	−115.17 (7)	O42B—N4B—C4B—C5B	−6.7 (4)
O42A—Cs1—O12A^ii^—C11A^ii^	63.7 (2)	C6A—C1A—C2A—N2A	180.0 (3)
O1W—Cs1—O11B^iii^—C11B^iii^	−43.5 (2)	C6A—C1A—C2A—C3A	−1.6 (4)
O2W—Cs1—O11B^iii^—C11B^iii^	30.0 (2)	C11A—C1A—C2A—N2A	0.8 (4)
O42A—Cs1—O11B^iii^—C11B^iii^	65.8 (2)	C11A—C1A—C2A—C3A	179.2 (2)
O1W—Cs1—O12A^iv^—Cs1^vii^	44.36 (6)	C2A—C1A—C6A—C5A	−0.1 (4)
O1W—Cs1—O12A^iv^—C11A^iv^	−134.0 (3)	C11A—C1A—C6A—C5A	179.1 (2)
O42A—Cs1—O12A^iv^—Cs1^vii^	−12.08 (5)	C2A—C1A—C11A—O11A	179.7 (2)
O42A—Cs1—O12A^iv^—C11A^iv^	169.6 (3)	C2A—C1A—C11A—O12A	−0.4 (4)
O1W—Cs1—O41B^iv^—N4B^iv^	−117.3 (3)	C6A—C1A—C11A—O11A	0.5 (4)
O2W—Cs1—O41B^iv^—N4B^iv^	−14.2 (3)	C6A—C1A—C11A—O12A	−179.6 (3)
O42A—Cs1—O41B^iv^—N4B^iv^	−90.9 (3)	C6B—C1B—C2B—N2B	179.9 (3)
O2W—Cs2—O1W—Cs1	4.49 (7)	C6B—C1B—C2B—C3B	1.1 (4)
O2W—Cs2—O1W—Cs1^vii^	−78.23 (13)	C11B—C1B—C2B—N2B	0.1 (4)
O41A—Cs2—O1W—Cs1	68.91 (6)	C11B—C1B—C2B—C3B	−178.6 (3)
O41A—Cs2—O1W—Cs1^vii^	−13.81 (11)	C2B—C1B—C6B—C5B	1.0 (4)
N2B—Cs2—O1W—Cs1	138.88 (6)	C11B—C1B—C6B—C5B	−179.2 (3)
N2B—Cs2—O1W—Cs1^vii^	56.16 (14)	C2B—C1B—C11B—O11B	177.5 (3)
O42B^v^—Cs2—O1W—Cs1	38.99 (17)	C2B—C1B—C11B—O12B	−3.9 (4)
O42B^v^—Cs2—O1W—Cs1^vii^	−43.7 (2)	C6B—C1B—C11B—O11B	−2.2 (4)
O12B^vi^—Cs2—O1W—Cs1	−126.67 (7)	C6B—C1B—C11B—O12B	176.3 (3)
O12B^vi^—Cs2—O1W—Cs1^vii^	150.61 (14)	N2A—C2A—C3A—C4A	−179.3 (3)
O42B^iv^—Cs2—O1W—Cs1	−62.41 (7)	C1A—C2A—C3A—C4A	2.2 (4)
O42B^iv^—Cs2—O1W—Cs1^vii^	−145.14 (12)	N2B—C2B—C3B—C4B	179.1 (3)
O1W—Cs2—O2W—Cs1	−4.31 (7)	C1B—C2B—C3B—C4B	−2.2 (4)
O41A—Cs2—O2W—Cs1	−71.86 (7)	C2A—C3A—C4A—N4A	177.3 (2)
N2B—Cs2—O2W—Cs1	−129.46 (7)	C2A—C3A—C4A—C5A	−1.1 (4)
O42B^v^—Cs2—O2W—Cs1	−168.65 (9)	C2B—C3B—C4B—N4B	−178.2 (2)
O12B^vi^—Cs2—O2W—Cs1	60.98 (10)	C2B—C3B—C4B—C5B	1.2 (4)
O42B^iv^—Cs2—O2W—Cs1	92.29 (7)	N4A—C4A—C5A—C6A	−179.1 (2)
O1W—Cs2—O41A—N4A	16.2 (2)	C3A—C4A—C5A—C6A	−0.7 (4)
O2W—Cs2—O41A—N4A	121.7 (2)	N4B—C4B—C5B—C6B	−179.7 (2)
N2B—Cs2—O41A—N4A	−102.1 (2)	C3B—C4B—C5B—C6B	1.0 (4)
O42B^v^—Cs2—O41A—N4A	−176.1 (2)	C4A—C5A—C6A—C1A	1.3 (4)
O12B^vi^—Cs2—O41A—N4A	−4.7 (3)	C4B—C5B—C6B—C1B	−2.1 (4)
O42B^iv^—Cs2—O41A—N4A	102.0 (2)		

Symmetry codes: (i) −*x*+3/2, *y*−1/2, −*z*+3/2; (ii) −*x*+1, −*y*, −*z*+1; (iii) −*x*+2, −*y*, −*z*+1; (iv) *x*+1/2, −*y*+1/2, *z*+1/2; (v) −*x*+3/2, *y*−1/2, −*z*+1/2; (vi) −*x*+2, −*y*+1, −*z*+1; (vii) −*x*+3/2, *y*+1/2, −*z*+3/2; (viii) *x*−1/2, −*y*+1/2, *z*−1/2; (ix) −*x*+3/2, *y*+1/2, −*z*+1/2.

### Hydrogen-bond geometry (Å, °)

**Table:**

*D*—H···*A*	*D*—H	H···*A*	*D*···*A*	*D*—H···*A*
N2A—H22A···O11B^v^	0.88 (3)	2.35 (3)	3.132 (3)	148 (3)
N2A—H21A···O12A	0.86 (4)	2.06 (4)	2.685 (4)	129 (3)
N2B—H21B···O11A^x^	0.90 (3)	2.08 (3)	2.848 (3)	143 (3)
N2B—H22B···O12B	0.82 (3)	2.04 (3)	2.657 (4)	131 (3)
O1W—H11W···O11B^vi^	0.90 (5)	1.88 (4)	2.768 (3)	169 (3)
O1W—H12W···O12A^x^	0.85 (4)	1.99 (4)	2.839 (3)	180 (5)
O2W—H21W···O11A^ii^	0.85 (4)	2.01 (4)	2.851 (4)	179 (6)
O2W—H22W···O12B^iii^	0.81 (4)	1.96 (4)	2.769 (4)	172 (4)
C6A—H6A···O11A	0.93	2.41	2.762 (3)	102
C6B—H6B···O11B	0.93	2.40	2.746 (3)	102

Symmetry codes: (v) −*x*+3/2, *y*−1/2, −*z*+1/2; (x) −*x*+1, −*y*+1, −*z*+1; (vi) −*x*+2, −*y*+1, −*z*+1; (ii) −*x*+1, −*y*, −*z*+1; (iii) −*x*+2, −*y*, −*z*+1.
